# The Role of Protein Persulfidation in Brain Aging and Neurodegeneration

**DOI:** 10.3389/fnagi.2021.674135

**Published:** 2021-06-23

**Authors:** Dunja Petrovic, Emilia Kouroussis, Thibaut Vignane, Milos R. Filipovic

**Affiliations:** Leibniz-Institut für Analytische Wissenschaften – ISAS - e.V., Dortmund, Germany

**Keywords:** persulfidation, neurodegenerative disease, aging, hydrogen sulfide, redox signaling

## Abstract

Hydrogen sulfide (H_2_S), originally considered a toxic gas, is now a recognized gasotransmitter. Numerous studies have revealed the role of H_2_S as a redox signaling molecule that controls important physiological/pathophysiological functions. The underlying mechanism postulated to serve as an explanation of these effects is protein persulfidation (P-SSH, also known as *S*-sulfhydration), an oxidative posttranslational modification of cysteine thiols. Protein persulfidation has remained understudied due to its instability and chemical reactivity similar to other cysteine modifications, making it very difficult to selectively label. Recent developments of persulfide labeling techniques have started unraveling the role of this modification in (patho)physiology. PSSH levels are important for the cellular defense against oxidative injury, albeit they decrease with aging, leaving proteins vulnerable to oxidative damage. Aging is one of the main risk factors for many neurodegenerative diseases. Persulfidation has been shown to be dysregulated in Parkinson's, Alzheimer's, Huntington's disease, and Spinocerebellar ataxia 3. This article reviews the latest discoveries that link protein persulfidation, aging and neurodegeneration, and provides future directions for this research field that could result in development of targeted drug design.

## Introduction

Hydrogen sulfide (H_2_S) is a small colorless gas that has sparked large controversy over the past two decades. Before the discovery that eukaryotes synthesize H_2_S and the recognition that it has a physiological purpose, for hundreds of years, H_2_S was viewed solely as a toxic gas released into the atmosphere by volcanic eruptions and utilized by bacteria and microbes. However, it was H_2_S that was used, together with cyanide and UV-light, to synthesize the building blocks of life such as RNA, lipids and nucleic acids (Patel et al., 2015) and early life forms thrived in H_2_S-rich environment for hundreds of millions of years (Olson and Straub, 2016). The recognition of the physiological importance of H_2_S started to emerge from the first report by Abe and Kimura, identifying that H_2_S is a neurological modulator in the brain (Abe and Kimura, 1996), stimulating a productive two decades of research.

**Figure 1:**
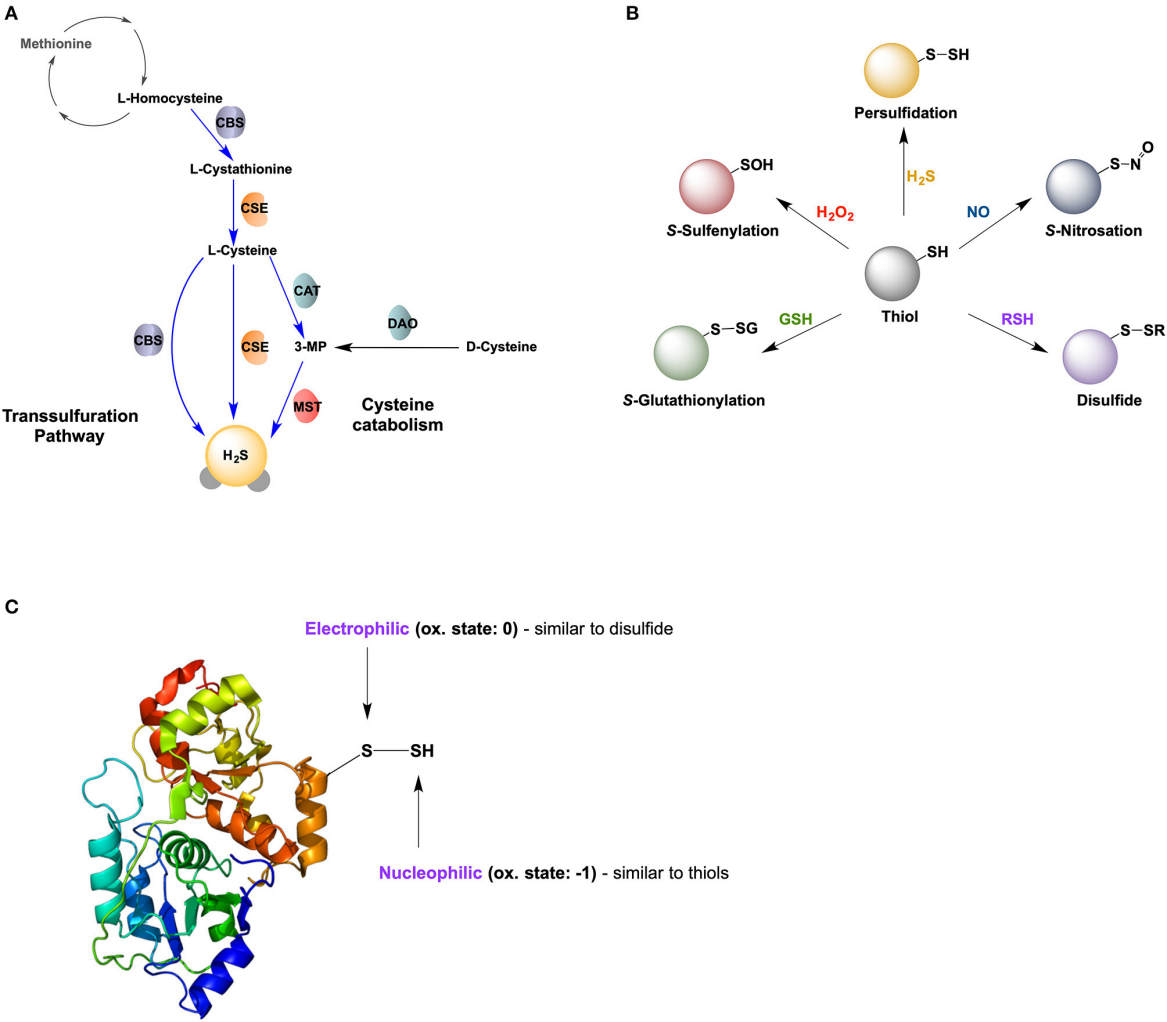
Hydrogen sulfide and protein persulfidation. **(A)** Biosynthesis of H_2_S *via* transsulfuration pathway and cysteine catabolism. CBS: cystathionine beta synthase; CSE, cystathionine gamma lyase; CAT, cysteine aminotransferase; DAO, D-amino acid oxidase; MST, mercaptopyruvate sulfur transferase. **(B)** Reversible posttranslational modifications of cysteine. **(C)** Dual chemical nature of protein persulfides, with one sulfur atom being electrophilic and the other nucleophilic.

There are three main enzymes involved in H_2_S formation. Two of the enzymes are pyridoxal 5′-phosphate (PLP)-dependent enzymes, cystathionine β-synthase (CBS), and cystathionine γ-lyase (CSE; also known as CTH) important for the transsulfuration pathway. These enzymes are predominantly located in the cytosol; however, their presence in other compartments, such as the nucleus and mitochondria, has been reported (Kabil et al., 2006; Fu et al., 2012; Teng et al., 2013). The third enzyme is the PLP-independent, 3-mercaptopyruvate sulfurtransferase (MST; also known as MPST), located in the mitochondria and the cytoplasm (Figure 1A) (Nagahara et al., 1998). In the central nervous system (CNS), CBS is predominantly expressed in glial cells, while CSE is the main contributor of H_2_S production in neurons. MST is widely distributed in all cell types (Paul et al., 2014; Zivanovic et al., 2019).

The reactivity of H_2_S in biological systems can be divided into three groups: (i) reaction with/scavenging of reactive oxygen and reactive nitrogen species (ROS and RNS); (ii) binding to and/or subsequent redox reactions with metal centers; and (iii) reaction with proteins, herein called persulfidation (alternatively, *S*-sulfhydration) (Filipovic et al., 2018).

Numerous physiological functions have been shown to exclusively or partly be regulated by H_2_S, some of which being vasodilation (Yang et al., 2008; Szijártó et al., 2018), neurotransmission (Abe and Kimura, 1996), angiogenesis (Szabó and Papapetropoulos, 2011), inflammation (Whiteman and Winyard, 2011), and hypoxia sensing (Peng et al., 2014). Additionally, H_2_S has been shown to induce a suspended animation-like state in mice (Blackstone et al., 2005). Furthermore, H_2_S shows a tremendous pharmacological potential (Szabõ, 2007; Wallace and Wang, 2015); with a quick search on Pubmed suggesting that H_2_S has a potential of curing almost every disease. Several pharmacological donors of H_2_S have also been developed with hope of their eventual use in disease treatment (Wallace and Wang, 2015; Whiteman et al., 2015), but the question of how H_2_S acts to alleviate all of the aforementioned diseases remains a hot topic of research.

## Protein Persulfidation

### Persulfide Biochemistry

Snyder's group proposed that the main mechanism for H_2_S signaling is a new oxidative post-translational modification of protein cysteine residues (P-SH), S-sulfhydration (or persulfidation, P-SSH) (Mustafa et al., 2009; Paul and Snyder, 2012, 2015). Persulfidation has been proposed to represent a new type of redox-switch reaction responsible for the regulation of protein structure and function (Figure 1B), alongside *S*-nitrosation (P-SNO), *S*-glutahtionylation (P-SSG), and *S*-sulfenylation (P-SOH).

Protein persulfidation has become increasingly recognized as the main mechanism by which H_2_S controls cellular functions. The persulfide group is a type of unsymmetrical disulfide, bearing two sulfur atoms with different properties (Figure 1C). Its inner sulfur; P-*S*SH is considered a sulfane sulfur with an oxidation state of 0, having a slightly electrophilic nature (thus, susceptible to nucleophilic attack) (Filipovic, 2015; Filipovic et al., 2018). However, its outer sulfur; P-S*S*H, has an oxidation state of−1 making it nucleophilic (thus, can react with electrophiles). P-SSH is ionisable and acidic, existing predominantly in its anionic form, P-SS^−^ at physiological pH 7.4 (Cuevasanta et al., 2015). Its fully ionized nature, coupled with an alpha effect from its adjacent sulfur, makes it a much stronger nucleophile (and thus more reactive) compared to its corresponding thiol (Cuevasanta et al., 2015; Filipovic et al., 2018). The nucleophilicity of persulfides renders them reactive to 1- and 2- electron oxidants (Cuevasanta et al., 2017; Filipovic et al., 2018).

One common misconception is that persulfides are formed *via* a direct reaction of H_2_S and a cysteine thiolate. However, this reaction is thermodynamically unfavorable and the effects claimed to be by “direct” protein persulfidation produced by treating proteins with H_2_S solutions have been assigned to the impurities in those solutions (Kimura et al., 2013; Wedmann et al., 2014; Zhang et al., 2014). The main mechanisms by which persulfides are non-enzymatically formed are through reactions of H_2_S with oxidized cysteines, such as P-SOH (Cuevasanta et al., 2015; Zivanovic et al., 2019), disulfides (Cuevasanta et al., 2015; Vasas et al., 2015), or in reactions of cysteine residues with sulfide radicals (Vitvitsky et al., 2018), polysulfides (Greiner et al., 2013), and other persulfides (so called transpersulfidation) (Ida et al., 2014). In addition, metalloproteins with iron or zinc in the active site could serve as catalysts for persulfide formation (Vitvitsky et al., 2018; Lange et al., 2019).

Persulfidation is considered to be a reversible modification and given the increasing evidence of its prominent signaling role, its endogenous removal is essential. Two parallel studies showed that the thioredoxin/thioredoxin reductase system efficiently reduces persulfides restoring the cysteine residue (Dóka et al., 2016; Wedmann et al., 2016). This “depersulfidase” activity has been observed in both cells and humans.

### Persulfide Detection

**Figure 2:**
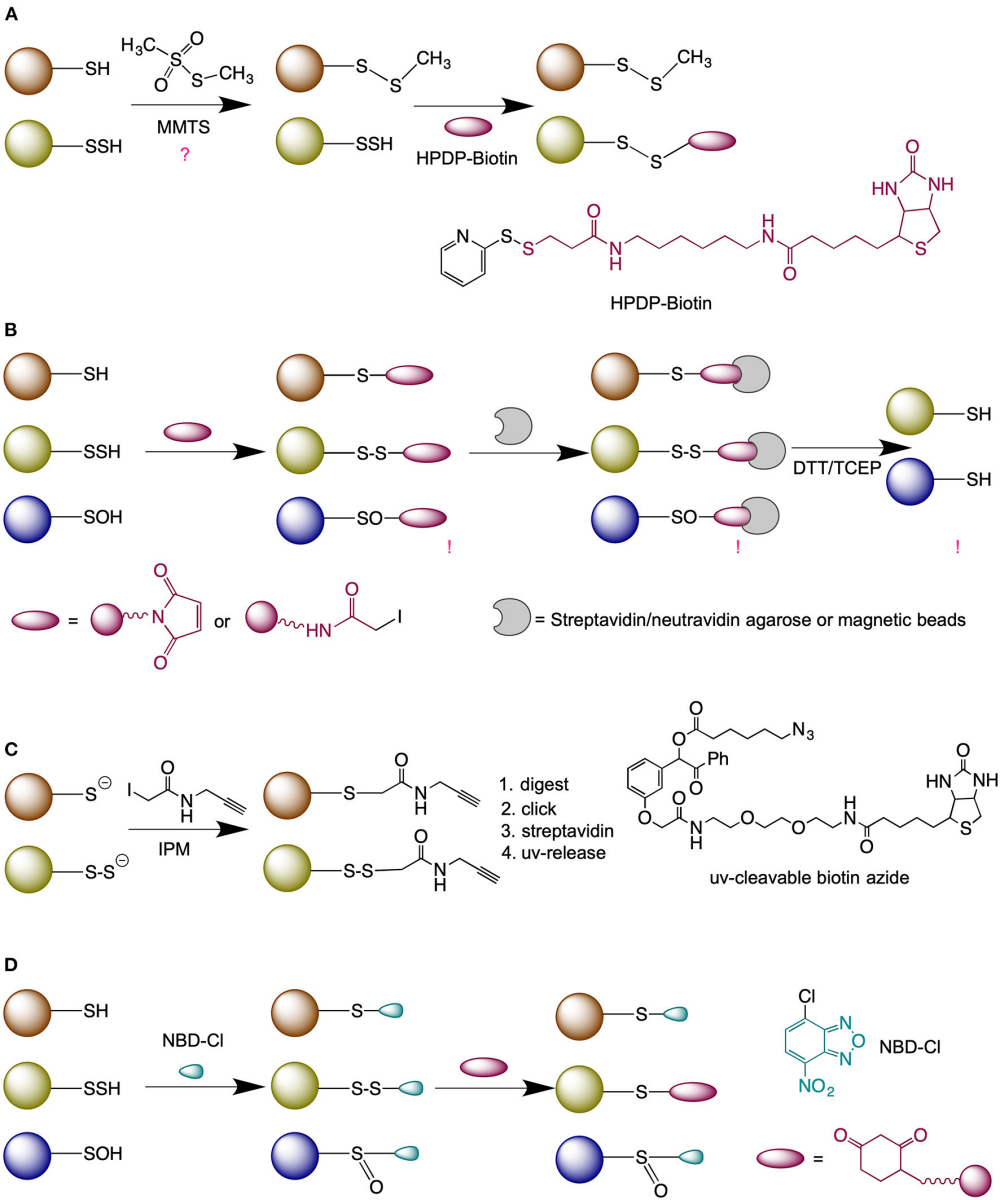
Methods for persulfide labeling. **(A)** Modified-biotin switch assay. **(B)** Method based on thiol blocking (either with N-ethyl maleimide or iodoacetmide) followed by the reduction with dithiothreitol (DTT) or tris(2-carboxyethyl)phosphine (TCEP). **(C)** Low-pH quantitative thiol reactivity profiling approach. **(D)** Dimedone-switch method for persulfide labeling. NBD-Cl, 4-Chloro-7-nitrobenzofurazan.

Although persulfides might appear as “one sulfur away” from regular thiols, it is this exact feature that makes them very reactive (Yadav et al., 2016) and difficult to label, which explains why this field still remains understudied despite persulfides widespread distribution. Several methods for persulfide detection have been proposed (Figure 2).

The first method described for the detection of persulfides was the modified biotin switch method (Mustafa et al., 2009) (Figure 2A). However, the selectivity of this approach was questioned due to the fact that persulfides are more reactive than thiols (Cuevasanta et al., 2015) and can readily react with methanethiosulfonate, as demonstrated by Pan and Carroll (Pan and Carroll, 2013).

The blocking of P-SSH with electrophiles, followed by its reduction was proposed originally by Snyder's group. The authors used fluorescently labeled maleimide to block thiols and persulfides and by comparing the intensity of the signal obtained with or without dithiothreitol (DTT) they calculated the yield of P-SSH (Sen et al., 2012) (Figure 2B). A modification of this approach was used by Cuevasanta et al. (2015) and Gao et al. (2015) to detect persulfides by Mass spectrometry (MS), and other modifications have been also reported (Dóka et al., 2016; Longen et al., 2016). While the approach is reliable when working with purified proteins, the method suffers from the lack of selectivity when applied on cell extracts (Fan et al., 2020).

An improvement to this approach and currently the only “direct” method for persulfide detection has been recently proposed by Yang's group (Fu et al., 2020), known as low-pH Quantitative Thiol Reactivity Profiling (QTRP). Alkylation is performed at a low pH to keep persulfides fully deprotonated and highly reactive, and the majority of free thiols protonated and less reactive. Using click chemistry and UV-cleavable biotinylated probes for peptide release, the authors cleverly avoided the reduction step and directly compared the *m/z* of peptides whilst taking into account the presence of additional sulfur in persulfide-containing peptides (Figure 2C).

The Tag-switch method proposed by Zhang et al. (2014) is based on a different chemical approach where thiols and persulfides are blocked with an aromatic thiol blocking reagent. In the case of persulfides this results in the formation of an activated disulfide bond; with different properties to endogenous disulfides and readily susceptible to specific nucleophiles tags (Zhang et al., 2014; Yadav et al., 2016; Aroca et al., 2017). An improvement of this method was recently published, named the Dimedone-switch method (Zivanovic et al., 2019) where commercially available dimedone-based probes were used as the nucleophilic tags (Figure 2D). The method proved to be very robust and versatile for any persulfide-detecting use.

Since the (bio)chemistry of H_2_S and protein persulfidation has been covered elsewhere (Filipovic, 2015; Paul and Snyder, 2015; Cuevasanta et al., 2017; Filipovic et al., 2018) in much more detail, we will focus on providing an overview of the role of protein persulfidation in the aging of the brain, with particularly emphasis on its role(s) in neurodegeneration.

## Relationship Between H_2_S/Protein Persulfidation and Aging

**Figure 3:**
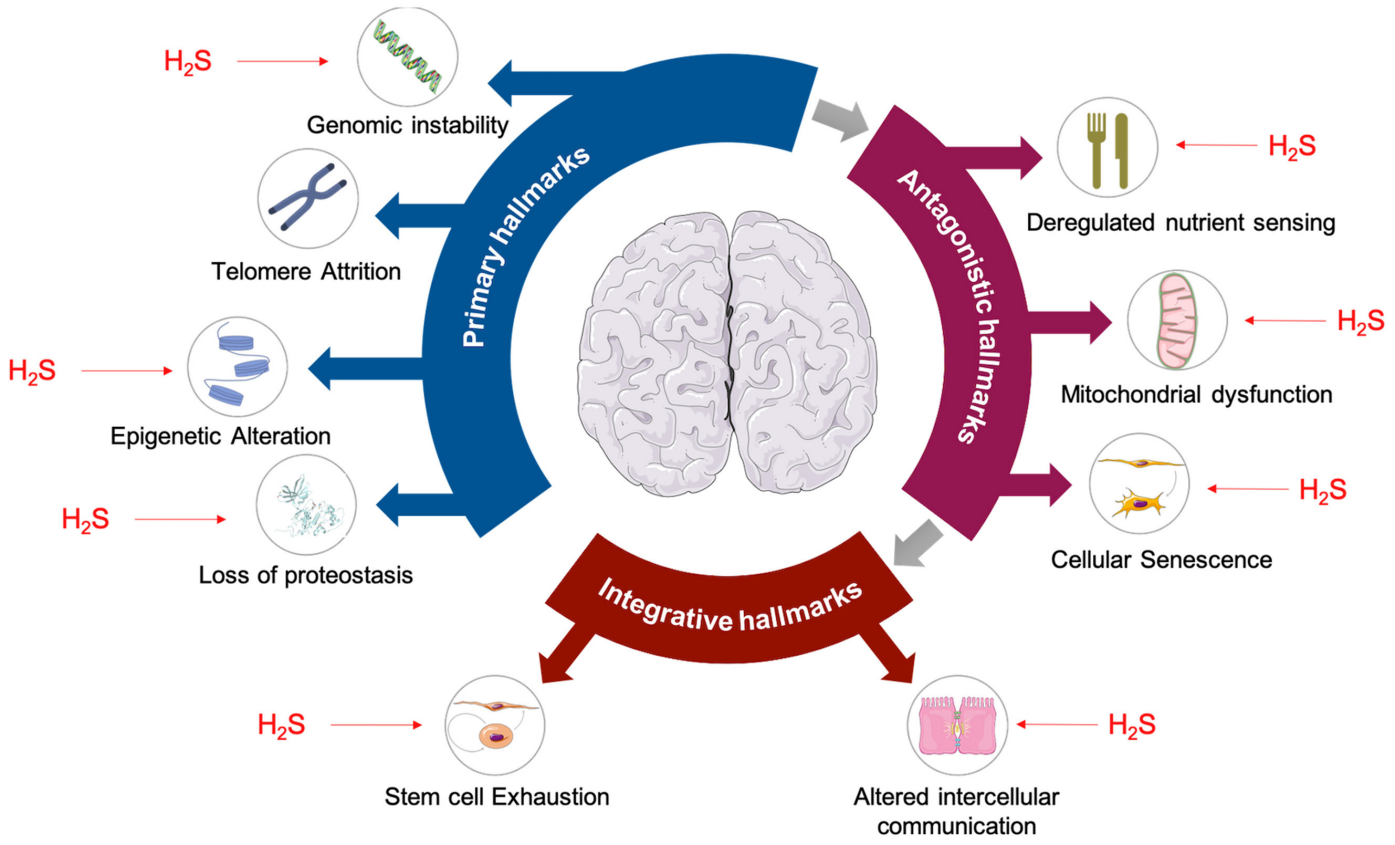
Hallmarks of aging are affected by H_2_S. Primary hallmarks of aging shown to be affected by H_2_S are: genomic instability (Szczesny et al., 2014; Zhao et al., 2014), epigenetic alterations (Suo et al., 2013; Rios et al., 2015), and loss of proteostasis (Liu et al., 2013; Fawcett et al., 2015). Antagonistic hallmarks of aging such as deregulated nutrient sensing (Xue et al., 2013; Ohno et al., 2015), mitochondrial dysfunction (Elrod et al., 2007; Sun et al., 2012), and cellular senescence (Yang et al., 2013; Zheng et al., 2014) are also affected by H_2_S. Finally, H_2_S is also known to have effects on stem cell exhaustion (Liu et al., 2014a,b) and intracellular communication (Munaron et al., 2013), which are considered to be integrative hallmarks of aging.

The aging process is a progressive loss of physiological function which emerges when an organism grows older. This process results from a time-dependant accumulation of cellular damage leading to a gradual loss of function at the molecular, cellular, tissue, and organismal level. Nine cellular hallmarks of aging have been defined (López-Otín et al., 2013) that can be clustered into three distinct but interconnected sub-categories: (i) the primary hallmarks, which are the primary causes of cellular damage; (ii) the antagonistic hallmarks, involved in the compensatory system of response to cellular damage but can also be deleterious; and (iii) the integrative hallmarks that are the result of the two previous categories and are the late cellular and tissue response to the functional decline associated with aging. H_2_S has been shown to affect 8 out of 9 aging hallmarks (Zhang et al., 2013; Perridon et al., 2016); some examples being the prevention of genomic instability (by modifying MEK/ERK pathway which leads to Poly(ADP-ribose) polymerase 1 activation and DNA repair mechanisms promotion) (Zhao et al., 2014) and prevention of epigenetic alteration (by modulating Sirtuin 1, one of the three histone deacetylases involved in the regulation of longevity and/or healthy aging mammals) (Du et al., 2019) (Figure 3).

Two independent large-scale proteomic analyses revealed that CSE protein levels steadily decline as *C. elegans* age (Walther et al., 2015; Narayan et al., 2016). Conversely, a recent comprehensive analysis of 17 known lifespan-extending interventions in mice, at the level of gene expression, identified CSE as a common denominator that is overexpressed (Tyshkovskiy et al., 2019).

Exposure to an H_2_S- containing atmosphere significantly prolongs lifespan in wild type (WT) *C. elegans* without affecting its physiology. The ability of H_2_S to induce a suspended animation-like state in mice (non-hibernating animals) (Blackstone et al., 2005) and the beneficial effects of H_2_S on lifespan (Miller and Roth, 2007), originally demonstrated by Roth's group, created hope in designing ways to slow down aging and/or even putting humans into a hibernation-like state (Asfar et al., 2014). Although not as profound, similar results were obtained using the slow-releasing H_2_S donor GYY4137 to which mimic physiological concentrations of H_2_S more closely (Qabazard et al., 2014); not only was the lifespan extended, but age-dependent changes were delayed in animals treated with GYY4137. Treatment with thiosulfate, which mimics beneficial effects of H_2_S, had a positive effect on *C. elegans* lifespan and correlated with high persulfidation levels in those animals (Zivanovic et al., 2019). Endogenous H_2_S production also proved to be essential for lifespan extension in germline-deficient *C. elegans* mutants, where reduced transsulfuration activity caused by the knockdown of the *cbs-1* gene significantly shortened the lifespan of germline-deficient mutants compared to WT (Wei and Kenyon, 2016).

Furthermore, CSE deficient mouse embryonic fibroblasts (MEF) have shown higher levels of oxidative stress in earlier passages and premature cell senescence in comparison with WT MEF. Oxidative stress resistance is dependent on the nuclear translocation of the antioxidant transcription factor Nrf2, which is enabled by persulfidation of its negative regulator Keap-1. Therefore, reduced H_2_S production results in decreased Nrf2 activity and impaired antioxidant response in CSE deficient MEF cells (Yang et al., 2013).

One of the most reliable aspects of aging in mammals is vascular decline and impairment of angiogenesis, which significantly contributes to deterioration of human health with age and the development of age-related cardiovascular diseases. A recent study on aging mice displayed the importance of H_2_S in restoring angiogenic potential later in life through sirtuin-dependent deacetylase SIRT-1 pathway. The role of H_2_S in cell senescence has also been demonstrated through the activation of the same pathway. The senescence of HUVEC cells (induced by oxidative stress or SIRT1 inhibition) was delayed upon H_2_S treatment by activating the SIRT1 protein (Das et al., 2018; Longchamp et al., 2018).

In fact, the beneficial effects of dietary restriction (DR), acknowledged as one of the most promising interventions to improve health and extend longevity in wide variety of species, have been linked to H_2_S. Seminal work by Mitchell's group showed, using numerous model organisms including yeast, worms, flies and rodents, that endogenous H_2_S levels increase upon different dietary restriction regimes (Hine et al., 2015). Furthermore, the lack of H_2_S producing enzymes in flies, worms, and rodents abolishes the positive effects of DR, while the overexpression of these enzymes mimic the effects of DR without any dietary intervention (Kabil et al., 2011; Hine et al., 2015).

## Persulfidation as an Evolutionarily Conserved Antiaging Mechanism

Questions to be answered are how can H_2_S extend lifespan and whether there can be a unifying mechanism that might, in part, explain other beneficial effects assigned to H_2_S? Even though the persulfidation of cysteine residues seems like a logical answer—how can persulfidation extend lifespan? As aforementioned, H_2_S cannot directly modify cysteine residues and an intermediary oxidation step is required. A drop in antioxidant defense mechanisms and increased ROS (mainly H_2_O_2_) production have long been postulated as key accelerators of aging (Balaban et al., 2005; Liochev, 2013; Sun et al., 2016). Our group recently proposed that persulfidation may be an evolutionary remnant of the times when life emerged in a sulfide-rich environment and that it represents the simplest way to resolve cysteine oxidation and protect proteins from oxidative damage (Zivanovic et al., 2019).

**Figure 4:**
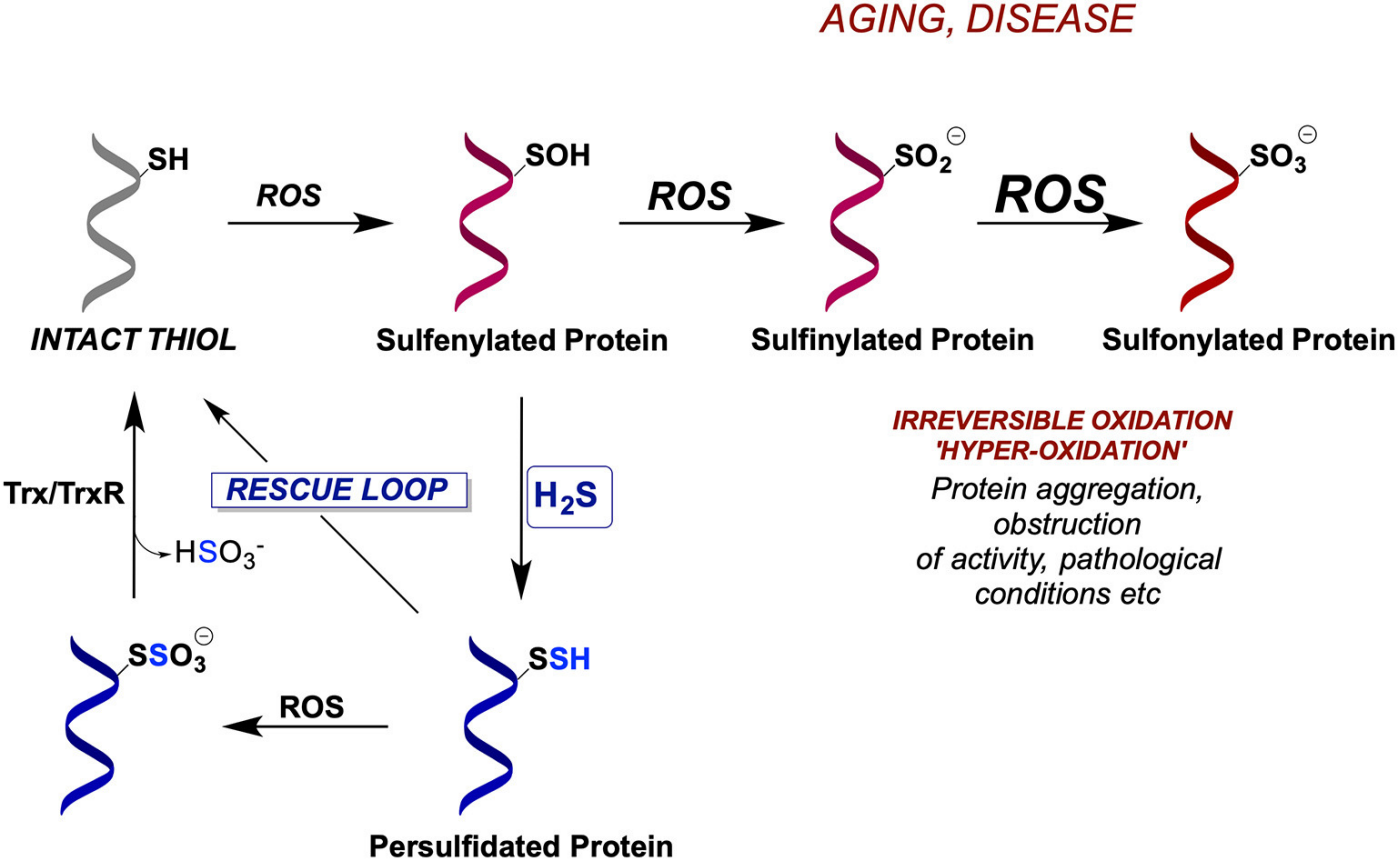
Evolutionarily conserved mechanism for cellular protection by protein persulfidation. Due to their increased nucleophilicity, persulfides are better scavengers of ROS than cysteines resulting in the formation of S-sulfonates, which could potentially be reduced by thioredoxin/thioredoxin reductase system (Trx/TrxR), restoring back the thiolates. In the absence of H_2_S, cysteine residues become irreversibly hyperoxidized leading to protein inactivation.

During oxidative stress cysteine residues get oxidized to sulfenic acids (Figure 4) representing an important signaling event for the cell to either start proliferating or to die (depending on the amount of H_2_O_2_) (Lo Conte and Carroll, 2013; Paulsen and Carroll, 2013). However, if left unreacted or exposed to further ROS, sulfenylated cysteines oxidize further to sulfinic (P-SO_2_H) and sulfonic acids (P-SO_3_H) (Chauvin and Pratt, 2017) which are generally considered irreversible [although some P-SO_2_H could be reduced back to thiols (Akter et al., 2018)] and if buried deep in protein pockets, P-SOH can be stabilized and not easily reachable for the reduction (back to P-SH) (Paulsen and Carroll, 2013). However, the reaction of P-SOH with H_2_S is ~600 times faster than with glutathione (Cuevasanta et al., 2015) and due to the small size of H_2_S it can reach deep into protein structures; indeed, we have observed increased P-SSH formation as a response to H_2_O_2_ stress (Cuevasanta et al., 2015; Wedmann et al., 2016). Once formed, P-SSH can be reduced back to P-SH by the thioredoxin (Trx/TrxR) system (Dóka et al., 2016; Wedmann et al., 2016). Therefore, not only does this mechanism preserve cysteine residues but it also represents a novel form of a redox control of protein function.

When oxidative stress persists (like in aging and many ROS-related diseases), P-SSH can act as better scavengers of ROS than P-SH, resulting in the formation of P-SSO_3_H. The existence of an S-S bond in P-SSH makes this group a potential target for Trx to restore it to its native thiolate (Figure 4), hence instead of the accumulation of damaged hyperoxidized proteins (containing P-SO_3_H), the overall structure, function and half-life of thiol-containing proteins can be preserved by their interim conversion to persulfides. We recently proved this mechanism showing that Trx is 2 orders of magnitude more efficient in cleaving cysteine S-sulfonate than cystine (Zivanovic et al., 2019).

Indeed, protein persulfidation was found to be conserved among different phyla and regna and was strongly dependent on the expression of the H_2_S producing enzymes, most notably CSE. Protein persulfidation also showed a closely intertwined nature with sulfenylation, serving as a redox switch and rescuing cysteines from further oxidative damage in cells exposed to endogenous or exogenous H_2_O_2_. More importantly, a global increase in P-SSH formation (either pharmacologically with H_2_S donors, or through dietary innervations, such as caloric restriction) proved to be protective against different ROS stressors and extended the lifespan of *C. elegans* (Zivanovic et al., 2019).

Somewhat surprisingly, protein persulfidation was found to decrease with aging in mice, rats, and humans. This effect was largely dependent on the decrease of some or all three H_2_S producing enzymes. In the case of rat brains, a progressive loss of protein persulfidation from the age of 6–24 months was caused by the profound decrease in CSE, CBS, and MST protein expression levels. Furthermore, a drop in P-SSH levels in fibroblasts, collected from the same donor at two different time points of his life was matched with an increase of P-SO_2_H levels (Zivanovic et al., 2019), further supporting the hypothesis of the overall protective effects of P-SSH in aging.

## CSE → H_2_S → PSSH Axis in Neurodegeneration

Neurodegenerative diseases (ND) are characterized by a progressive damage to neurons that results in compromised cognitive and/or motor functions. One of the common characteristics of ND is its age-dependency; for instance both Alzheimer's and Parkinson's diseases affect 10 and 2% of the elderly population in the USA, respectively (Liu et al., 2017; Venkatachalam et al., 2020). Recent analysis of primate proteomes with different ND found that 16% of the analyzed proteins contained one or more cysteines unique to primates. Structural analysis of these proteins revealed that in a vast majority of the cases, these unique cysteine residues were surface-exposed, making them more susceptible to oxidation. The authors identified this group of proteins as “primate differential redoxome” showing that it contains multiple deterministic and susceptibility factors of major ND (Venkatachalam et al., 2020). In the light of the above-mentioned facts that protein persulfidation acts as a general protective mechanism, it is tempting to speculate that there might be a causal link between aging-induced P-SSH decline and ND.

A common characteristic reported in the literature, found in even unrelated ND, is the loss/decrease of CSE which has been observed in human samples of Parkinson's, Huntington's, Alzheimer, and spinocerebellar ataxia 3 diseases, as well as in corresponding animal disease models (Vandiver et al., 2013; Paul et al., 2014; Snijder et al., 2015; Giovinazzo et al., 2021). A global decrease of protein persulfidation has also been observed for the latter three diseases (Snijder et al., 2015; Zivanovic et al., 2019; Giovinazzo et al., 2021). Besides a general protection, persulfidation could also represent a redox switch mechanism by which protein structure and/or function might be affected, and the search of these particular targets may pave the way to more targeted drug design toward ND therapeutics.

### Parkinson's Disease

**Figure 5:**
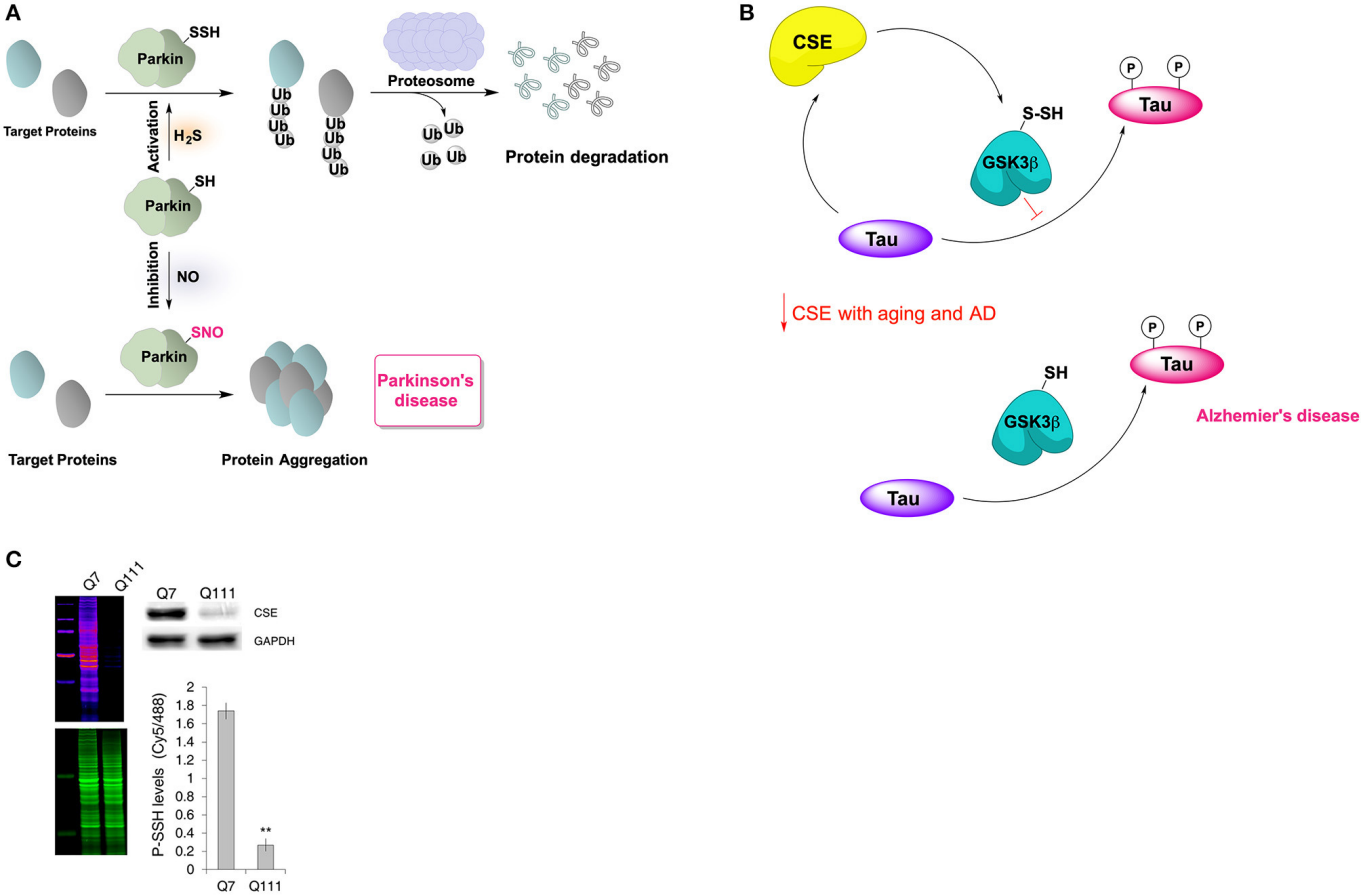
Neuroprotective effects of protein persulfidation. **(A)** Schematic representation of the regulatory role of H_2_S on catalytic activity of parkin. In healthy subjects, the E3 ubiquitin ligase, Parkin is persulfidated, which increases its enzymatic activity. This leads to ubiquitylation of diverse substrates, such as alpha-synuclein (a component of the Lewy bodies found in Parkinson's disease), and their subsequent proteasomal degradation. In patients with Parkinson's disease, parkin is S-nitrosylated with greatly decreased catalytic activity, resulting in protein aggregation, accumulation of toxic proteins and cell death. **(B)** Cystathionine gamma lyase (CSE) interacts with tau protein which results in allosteric modulation of its activity resulting in higher H_2_S production and consequently persulfidation of glycogen synthase kinase 3 beta (GSK3β). Persulfidation of GSK3β inhibits its kinase activity resulting in less tau phosphorylation. With aging, or in Alzheimer's disease, CSE levels decrease making the GSK3β predominantly present in thiolate form which results in increased tau phosphorylation and aggregation. **(C)** Protein persulfidation levels are almost entirely diminished in striatal cells expressing huntingtin with 111 polyQ repeats. This decrease of PSSH levels is caused by a big reduction in CSE levels. Taken from Zivanovic et al. (2019).

Parkinson's disease (PD) is a neurodegenerative disease caused by the death of dopamine-generating cells in the *substantia nigra* and one of the proteins considered responsible for this cell death is parkin (Shulman et al., 2011). Parkin is an E3 ubiquitin ligase with reactive cysteine residues that are susceptible to oxidative posttranslational modifications and modulate the protein's activity. For example, *S*-nitrosation of parkin inhibits its E3 ubiquitin ligase activity (Chung et al., 2004). It has been demonstrated recently that parkin can also be persulfidated at C59, C95, and C182 (Vandiver et al., 2013). The persulfidation of parkin, demonstrated even under basal conditions, leads to enzyme activation and clearance of damaged proteins (Figure 5A) (Vandiver et al., 2013). This has been observed *in vivo*, in samples from PD patients, which contained lower levels of persulfidated parkin, but increased levels of nitrosylated parkin (Vandiver et al., 2013).

In addition to parkin, the PD protein DJ-1 (also known as PARK7) is also known to be involved in the pathogenesis of PD. DJ-1 (also known as PARK7) undergoes oxidation to a sulfinic (Akter et al., 2018) and sulfonic acid (Fernandez-Caggiano et al., 2016), particularly at its Cys106. Sulfonylation of DJ-1 controls its intracellular localization and is implicated in protection against neuronal cell death (Canet-Avilés et al., 2004). We recently showed that DJ-1 undergoes protein persulfidation, which controls the levels of hyperoxidized form of this protein (Zivanovic et al., 2019). Whilst these data suggest that the use of H_2_S releasing drugs could have potential in preventing PD progression they also warrant for more detailed studies on animal models where the pharmacological role of H_2_S in PD could be assessed.

### Alzheimer's Disease

Levels of H_2_S in the brain of Alzheimer's disease (AD) patients have been found to be considerably low when compared to the healthy individuals (Eto et al., 2002; Giuliani et al., 2013) while pre-treatment with NaHS improved learning and memory deficits in rat AD model (Xuan et al., 2012). We recently showed that the tau protein binds to CSE increasing its H_2_S producing activity. In addition, we observed that H_2_S causes persulfidation of glycogen synthase kinase 3β at C218, resulting in the loss of the enzyme's activity and diminishing tau phosphorylation (Giovinazzo et al., 2021). A decrease of CSE levels in aging brain and consequently the decrease of GSK3β persulfidation will result in an accumulation of phosphorylated tau and tau aggregation (Figure 5B). Interestingly, the treatment of 3xTg-AD mice with GYY4137 restored global P-SSH levels and improved cognitive deficits.

In addition to GSK3β persulfidation, H_2_S exhibited an inhibitory role on the gene and protein expression of BACE-1 (beta-site APP cleaving enzyme-1), a major β-secretase involved in amyloid beta (Aβ) production (Zhang et al., 2011).

Further studies addressing the causal relationship between age-induced decrease of protein persulfidation and AD could help unraveling the potential drug targets for the H_2_S-releasing therapeutics but also identify eventual markers of this disease. Considering a high number of AD patients globally and documented H_2_S releasing effects of some natural products (Pluth et al., 2015), it could be worth testing them as supplementary therapy for AD patients.

### Poly-Q Diseases

One of the most striking examples of how the dysregulation of the CSE → H_2_S → P-SSH axis could lead to ND, is that of CSE knockout mice exhibiting Huntington's disease-like phenotype. Inspiring work by Snyder and Paul has demonstrated that CSE mutant mice display neurologic abnormalities, such as hind limb clasping which resembles mouse models of HD. Most importantly, in the striatum of human HD patients CSE levels were reduced by 85–90%, with greater reductions being observed in patients that displayed more severe clinical manifestations of the disease (Paul et al., 2014).

Similar effects have been observed in another polyglutamine (polyQ) disease called spinocerebellar ataxia 3 (SCA3). In both a *D. melanogaster* model and humans the levels of CSE in the brain were reduced (Snijder et al., 2015) and we also showed that this results in diminished P-SSH levels. Similarly, striatal cells models of HD expressing huntingtin with 111 polyQ repeats displayed strikingly low global P-SSH levels (Figure 5C) (Zivanovic et al., 2019).

Paul, Snyder and co-workers observed that mutated huntingtin binds to transcription factor SP1, which is responsible for CSE expression, and that repressed ATF4 activity in HD further downregulates CSE levels (Paul et al., 2014; Sbodio et al., 2016). Treatment of HD cells with monensin, a Golgi stress inducer, resulted in activation of PERK-ATF4-CSE pathway and subsequent increase of H_2_S and protein persulfidation leading to the better resistance of those cells to oxidants (Sbodio et al., 2018). Similarly, in *D. melanogaster* model of SCA3, overexpression of CSE rescued the disease phenotype, as manifested by decreased eye degeneration (Snijder et al., 2015).

## Future Directions

The field of protein persulfidation is still very young but accumulating evidence suggests that this modification can be of great importance in understanding fundamental biological processes and in designing new therapeutics. There are few directions the field could be developing toward, in order to provide definitive answers about the role of protein persulfidation in neurodegeneration, some of which have been addressed in more details below.

### Persulfidome vs. Other Posttranslational Modifications in Brain

To what extent are effects of protein persulfidation a consequence of a global protection (as described in Figure 3) and to what a consequence of a structure/function change induced by PSSH? These questions could be addressed by systematic proteomic analysis. Unlike S-nitrosylation, whose role in neuronal signaling and neurodegeneration has been extensively studied (Uehara et al., 2006; Cho et al., 2009; Nakamura et al., 2013; Seneviratne et al., 2016), the persulfidome changes in the brain, under healthy or disease conditions, have not been addressed. With the development of new targeted proteomics methods for protein sulfenylation (Fu et al., 2019), sulfinylation (Akter et al., 2018), cysteine oxidation and persulfidation (Zivanovic et al., 2019; Fu et al., 2020) it would be of interest to correlate the changes of all these cysteine modifications and other such as S-nitrosylation in brain aging and different ND models. Recent proteomic findings suggest that protein S-nitrosylation increases with aging of the brain, which is opposite to what has been reported for protein persulfidation (Kartawy et al., 2020). The cross-talk between S-nitrosylation and P-SSH is also still an unanswered question (Filipovic et al., 2012), with examples like parkin where these two modifications have completely opposite effects on its activity (Chung et al., 2004; Vandiver et al., 2013).

Considering that many kinases and phosphatases have been reported to be persulfidated (Zivanovic et al., 2019; Fu et al., 2020), the effect(s) of P-SSH on protein phosphorylation should also be addressed. GSK3b is just one example of how protein persulfidation can control the phosphorylation of protein and prevent progression of the disease (Giovinazzo et al., 2021).

ND are characterized by the aggregation of many proteins that should normally undergo degradation *via* the two main catabolism pathways, the ubiquitin-proteasome system and the autophagy-lysosomal pathway (Le Guerroué and Youle, 2021). The effects of H_2_S on both of those pathways have been reported in the literature. Future studies, based on brain persulfidome analysis should identify the targets that control these pathways and address their role in disease progression paving the way for the development of innovative therapeutic strategies that will permit targeted redox control of the cell metabolism and delay aging and disease progression.

### Development of H_2_S Donors to Treat ND

The use of water-soluble sodium salt of GYY4137 improved motor and cognitive deficits in AD mouse model (Giovinazzo et al., 2021), while administration of NaHS once a day for 3 months ameliorated memory deficits in another study (Liu et al., 2016). However, most of the studies done to date use inorganic sulfide salts as a source of H_2_S and although they are good for proof-of-concept observation there is a need for a development of real H_2_S-releasing therapeutics. Several have entered clinical trials but mainly for treating inflammation (Wallace and Wang, 2015; Whiteman et al., 2015). Design of slow-releasing H_2_S donors that could pass blood-brain barrier and be delivered could prove useful in treating some of the ND and ameliorating the general healthspan. Particularly interesting could be mitochondria-targeted H_2_S donors (Le Trionnaire et al., 2014). For example, AP39 is known to generally improve mitochondrial bioenergetics (Gero et al., 2016; Etheridge et al., 2017; Fox et al., 2021) and supplementation of APP/PS1 mouse model of AD P39 improved mitochondrial dynamics, shifting from fission toward fusion, ameliorated their spatial memory deficits and reduced Aβ deposition in their brains (Zhao et al., 2016).

In addition to the development of H_2_S donors, the search for drugs that increase the expression of H_2_S producing enzymes could be another interesting direction to go.

## Author Contributions

All authors listed have made a substantial, direct and intellectual contribution to the work, and approved it for publication.

## Conflict of Interest

The authors declare that the research was conducted in the absence of any commercial or financial relationships that could be construed as a potential conflict of interest.
